# Unlocking the Subconscious Consumer Bias: A Survey on the Past, Present, and Future of Hybrid EEG Schemes in Neuromarketing

**DOI:** 10.3389/fnrgo.2021.672982

**Published:** 2021-05-17

**Authors:** Fotis P. Kalaganis, Kostas Georgiadis, Vangelis P. Oikonomou, Nikos A. Laskaris, Spiros Nikolopoulos, Ioannis Kompatsiaris

**Affiliations:** ^1^MKLab, Center for Research and Technology Hellas, Information Technologies Institute, Thessaloniki, Greece; ^2^Artificial Intelligence & Information Analysis Lab, Department of Informatics, School of Sciences, Aristotle University of Thessaloniki, Thessaloniki, Greece

**Keywords:** consumer neuroscience, neuromarketing, multimodal, hybrid, EEG, eye tracker, survey, review

## Abstract

Fueled by early success stories, the neuromarketing domain advanced rapidly during the last 10 years. As exciting new techniques were being adapted from medical research to the commercial domain, many neuroscientists and marketing practitioners have taken the chance to exploit them so as to uncover the answers of the most important marketing questions. Among the available neuroimaging technologies, electroencephalography (EEG) stands out as the less invasive and most affordable method. While not equally precise as other neuroimaging technologies in terms of spatial resolution, it can capture brain activity almost at the speed of cognition. Hence, EEG constitutes a favorable candidate for recording and subsequently decoding the consumers' brain activity. However, despite its wide use in neuromarketing, it cannot provide the complete picture alone. In order to overcome the limitations imposed by a single monitoring method, researchers focus on more holistic approaches. The exploitation of hybrid EEG schemes (e.g., combining EEG with eye-tracking, electrodermal activity, heart rate, and/or other) is ever growing and will hopefully allow neuromarketing to uncover consumers' behavior. Our survey revolves around last-decade hybrid neuromarketing schemes that involve EEG as the dominant modality. Beyond covering the relevant literature and state-of-the-art findings, we also provide future directions on the field, present the limitations that accompany each of the commonly employed monitoring methods and briefly discuss the omni-present ethical scepticizm related to neuromarketing.

## 1. Introduction

Neuromarketing is an evolving field that bridges the gap between consumer behavior studies and neuroscience. In a more strict sense,neuromarketing refers to the “application of neuroscience in the marketing field.” According to this definition, neuromarketing studies should include, among others, the direct use of neuroimaging technology in order to explore a consumer's response to specific marketing elements (products, packaging, advertising, etc.). This perspective has allowed “neuromarketing” to become the applied counterpart and a relative term to “consumer neuroscience.” Additionally, it mitigates one of the major challenges in the field, that is the conceptual tautology between conventional marketing and neuromarketing. In some sense conventional marketing and neuromarketing could be conflated, since typical marketing campaigns aim to evoke some kind of brain activity that will lead to a desired behavioral response (e.g., buying a product). However, this is not a particularly insightful perspective since it extends neuromarketing's focus into a very wide domain; hence, making its target rather vague.

Neuromarketing constitutes an important development in the field of understanding how the subconscious mind drives the consumers' decisions. Since its infancy stage in 2002 (Smidts, 2002) and after the controversy that initially governed the field (Brammer, 2004), neuromarketing is gaining rapid credibility with ever-increasing adoption rates among advertising and marketing professionals. The recent development in long-standing science and neuroimaging technology accompanied by the growth of advertising needs have led neuroscientists and marketers to a common ground that enables the bridging of neuroscience and marketing. In some cases, the measured cognitive activity, as imprinted by the neuroimaging technologies, may not be consciously perceived by the subject, constituting the recorded data more revealing than self-reporting on surveys and focus groups (Ozdemir and Koc, 2012).

Out of the neuroimaging methods currently available, electroencephalography (EEG) is the least invasive and most cost-effective. Although EEG may lack in terms of spatial resolution, compared to other neuroimaging technologies, such as fMRI and fNIRS, it can reliably capture brain activity changes over smaller increments of time. The aforementioned benefits make EEG a favorable candidate for recording and subsequently decoding the consumers' brain activity. Additionally, over the last years, research in neuroscience has led to significant improvements in terms of decoding the users' brain activity when captured by an EEG device. More specifically, EEG practitioners and neuroscientists have managed to uncover neural signatures capable of indicating cognitive aspects that are of particular interest in the context of marketing studies (Hakim and Levy, 2019). Therefore, this survey considers only studies that employ EEG recordings in the field of neuromarketing.

The key concept of employing EEG in neuromarketing is to measure processes, such as decision-making, reward processing, memory and attention, approach and withdrawal motivation, mental workload, and emotional processing, by means of identifying specific electrical brain activity signatures. Decision making refers to the process of gathering information, assessing alternative resolutions, and reaching to a decision (Brockmann and Anthony, 2002). Despite the wide variety of cognitive processes that drive consumer behavior, understanding decision making is probably the “holy-grail” of neuromarketing. Answering questions like “how does a consumer cope with various product alternatives based on subjectively perceived benefits, expenses, and taste?” seems to be the driving force that underlies every (neuro)marketing study. Since such a complicated question cannot be answered directly using existing technology, there have been efforts to exploit other cognitive processes that are considered as the crucial factors that modulate decision making.

When people interact with a stimulus (i.e., a product, a brand, an advertisement, etc.), they can be either attracted to it or not (approach-withdrawal) (Harlé and Sanfey, 2010). Attractive elements (i.e., an appealing product design, a preferred brand) can be perceived as rewarding stimuli within consumers' brains and may consequently trigger the psychological motivations that affect the purchase behavior (Samanez-Larkin and Knutson, 2014). While making decisions, such as whether to purchase a product or not, people invest effort to process information coming from both the external environment and personal experience (Soria-Oliver et al., 2017). In cognitive psychology, this process is referred to as mental workload. Even though people's processing capacity is limited, consumers are exposed to an enormous amount of information on a daily basis. As people do not consciously attend to every bit of information they are exposed to, paying attention to something implies that a mechanism of selecting information exists and prioritisez particular information elements over other. It is reasonable to assume that attention, perception and processing of incoming information has a profound influence on consumer behavior (Rolls, 2007). Contemporarily, emotions are considered as strong influence factors that drive consumer choices and are very important in the decision-making process (Lerner et al., 2015). Therefore, the neuromarketing community has put significant effort to uncover the brain activity signatures that are highly correlated with emotions, attention, approach-withdrawal motivation, mental workload, reward processing, and others.

**Figure 1:**
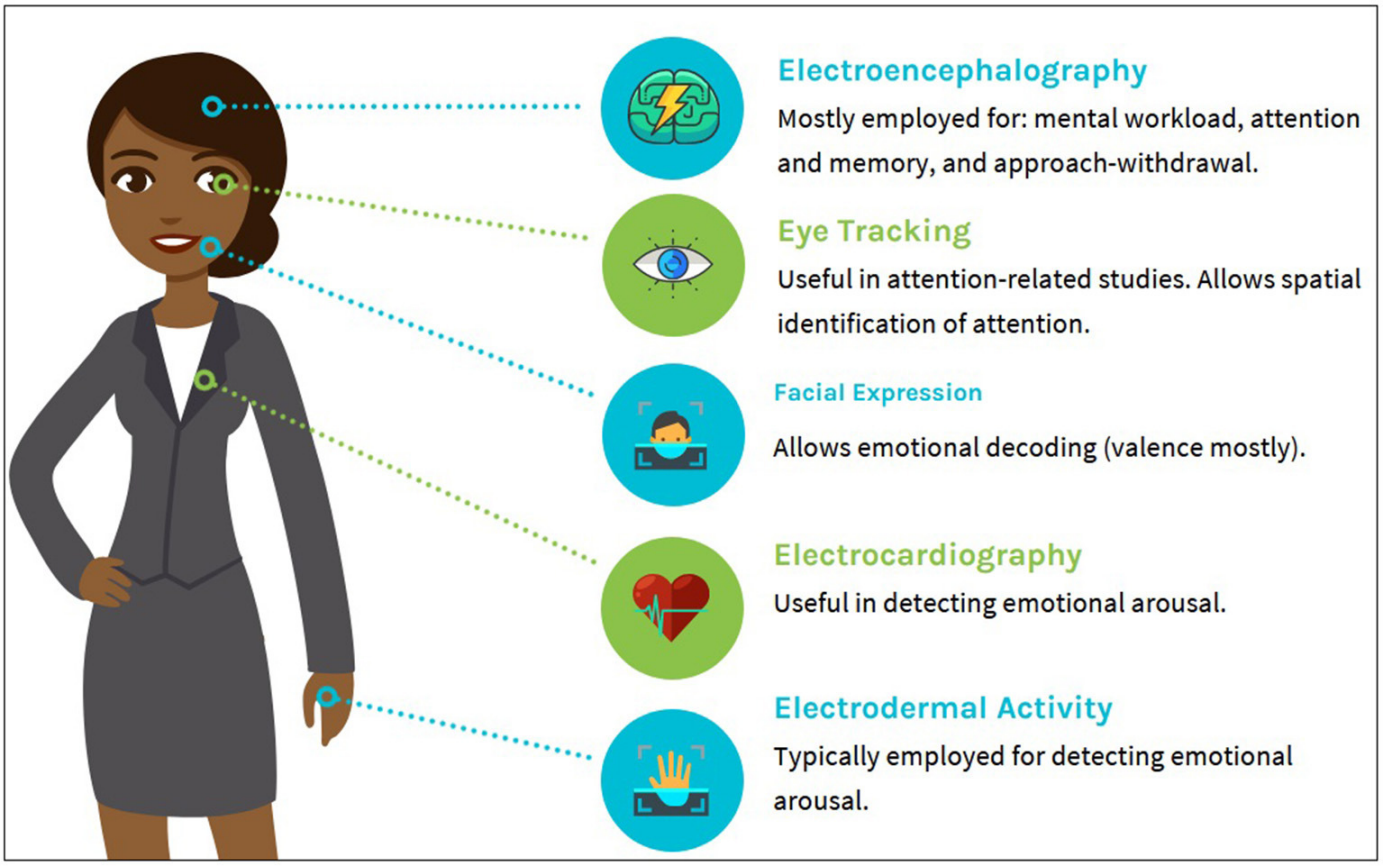
Most widely employed modalities and their corresponding use in neuromarketing.

While the aforementioned hold true it should be noted that, although EEG may provide valuable insights about the underlying neural processes of the consumer, it fails to give answer to more complex questions, such as “which part of the advertising fllyer captured the consumers' attention?” Attention is of course a cognitive process that can be assessed using EEG, but the objective and precise identification of its focus is a task that exceeds the range of possibilities carried by the EEG signals, commonly referred to as brainwaves. In order to get such delicate information, neuroimaging technologies may be complemented by various physiological monitoring tools to further boost the level of extracted information (Bercea, 2012). By exploiting hybrid neuroimaging recording schemes (e.g., combining EEG with eye-tracking, electrodermal activity, heart-rate, etc.), neuromarketing advances to a new era. Since physiological signal monitoring has become a popular practice in the marketing field, our survey emphasizes on hybrid neuromarketing schemes that combine EEG recordings with other types of physiological signals. Figure 1 summarizes the most commonly employed modalities in neuromarketing studies and their corresponding use.

Undeniably, neuromarketing is a growing field and several useful survey studies concern the employed tools (Bercea, 2012), the investigated neural processes (e.g., Bastiaansen et al., 2019), and even both of the aforementioned (Cherubino et al., 2019). However, to the best of our knowledge this is the first attempt that focuses on hybrid EEG neuromarketing schemes covering both the modalities and the corresponding literature. The present study aims to endow the reader with a solid understanding about the hybrid EEG-related methods and neurophysiological activity descriptors that have proven to be key components in the neuromarketing field. It is within our scope to cover not only the benefits, but also to address the major problems that may arise in such multimodal settings. Along with covering the relevant literature and exploring the state-of-the-art practices, potential future directions on the field are provided and the omni-present potential ethical issues linked to neuromarketing are discussed. We believe that our work will bring additional benefit to the field serving as a reference guide in the upcoming neuromarketing research.

## 2. Measuring the Physiological Responses

Although, the wide variety of physiological monitoring methods commonly used in neuromarketing can provide useful insights about consumers' responses to marketing stimuli, a single approach is not capable of providing the complete picture. In order to overcome the limitations imposed by a single monitoring method, researchers adopt on more holistic approaches. Thus, our study emphasizes on hybrid EEG schemes and this section presents, alongside the EEG, all the identified modalities employed in recent neuromarketing studies as complementary methods for investigating consumers' responses. Beyond explaining how each of the identified modalities works and what it can measure, we also provide information regarding its complementary attributes with respect to the EEG. Table 1 summarizes the main strengths and weaknesses of the most widely employed tools in neuromarketing research.

**Table 1:** Pros and Cons of the most commonly employed monitoring methods in neuromarketing.

**Modality**	**Pros**	**Cons**
Electroencephalography (EEG)	High temporal resolution	Low spatial resolution
	Useful for a wide variety of neural indicators	Cannot capture deep brain neural activity
	Modest technical requirements	Prone to artifacts
Eye tracking (ET)	Localization abilities	Eye movements interfere with EEG
	Useful for attention metrics	
	Pupil dilation is associated with cognitive processes	
Electrodermal activity (EDA)	Convenient and commercial	Measurements lag compared to the actual responses
	Indicates stress, emotional engagement, and other	
Electrocardiography (ECG)	Heart rate indicates arousal	Measurements are affected by physical exertion
Facial expression (FE)	Easy to interpret	Can be easily manipulated
	Reliable indicators of emotions	

### 2.1. Electroengephalography

Electroencephalography (EEG) is an electrophysiological monitoring method to record the electrical activity of the brain. EEG signals reflect the meso- and macroscopic neural dynamics produced by large populations of cortical neurons (Cohen, 2014). As a cognitive electrophysiology monitoring method, it allows researchers to investigate how cognitive functions, such as emotions, memory, perception, behavior monitoring/control and others, are supported or implemented by the electrical activity produced by populations of neurons. It is a non-invasive monitoring method, with the electrodes placed over the scalp. EEG measures voltage fluctuations (also referred to as brainwaves) resulting from ionic current within the neurons of the brain. These electrical signals are the means by which our brain passes information and synchronizes activity across its different anatomical regions. The fluctuations of the recorded electrical activity serve as indicators of changes in cognitive processing. Modern EEG equipment can take a snapshot of brain's electrical activity every 1–3 ms, providing greater temporal resolution than any other neuroimaging technology. Therefore, EEG is the most suitable for studying fast neural events in time like the ones emerging when someone watches a TV commercial.

In the context of neuromarketing, the analysis of EEG recordings typically involves, but is not limited to, (i) spectral analysis; where the frequency characteristics of the multivariate signal are studied, (ii) hemispheric asymmetry; where the activity stemming from one brain hemisphere is contrasted against the activity stemming from the other, and (iii) event-related potential analysis; where individual brain responses that occur as a direct aftereffect to particular stimuli are investigated and analyzed. Each of the aforementioned analyses emphasizes on a different aspect of EEG and is therefore governed by particular advantages and disadvantages with respect to answering neuromarketing questions.

Brain signals that stem naturally by the brain exhibit distinctive frequency characteristics. The frequency of the recorded activity is measured in Hertz (Hz) which expresses cycles per unit of time (second). Typically, the most dominant frequency which is present in brainwaves revolves around the 10 Hz. However, the strength of each frequency component varies with respect to different mental states, and also changes with the course of time and across different brain regions. Brainwaves are conventionally classified into distinct, non-overlapping, frequency bands each of which is associated with specific psychological/cognitive processes that are named after Greek letters:

- δ-delta (1–4 Hz): dominant in dreamless sleep
- θ-theta (4–8 Hz): associated with memory activation, conscious concentration
- α-alpha (8–12 Hz): dominant during relaxed states whereas suppressed under attentional stimuli, considered as the default brain's frequency
- β-beta (12–30 Hz): associated with active attention, alertness and reward expectation
- γ-gamma (>30 Hz): associated with information processing, learning, and emotional processing.

Among the various spectral descriptors, the hemispheric asymmetry has gained great popularity in the neuromarketing studies. Typical methodologies measure frequency band asymmetries (e.g., differences) between the left and right frontal region of the brain. This is attributed to the fact that this measurement has been connected to motivation of approach and withdrawal with respect to an element (e.g., a product) of attention. When someone is attracted by an element (i.e., approach state), the brain produces stronger (e.g., larger amplitude) beta and gamma brainwaves and weaker alpha brainwaves in the left-frontal hemisphere compared to the right, whereas the opposite trend is observed while being repelled away from it (i.e., withdrawal) (Briesemeister et al., 2013). More recently, Ramsøy et al. (2018) demonstrated that prefrontal asymmetry in the gamma frequency band was significantly related to subsequent “willingness to pay” responses. In principle, increased left-frontal activity may serve as an index of positive behavioral responses and right-frontal as an index of negative ones.

Beyond the spectral-related characteristics of EEG, temporal dynamics have a long tradition in EEG studies. One of the most promising studies with respect to temporal characteristics in neuromarketing concerns the study of event-related potentials (ERPs). ERPs refer to EEG changes that are both time and phase locked to sensory, motor or cognitive events. ERPs are typically obtained by averaging multiple EEG segments temporally locked to the event of interest. The amplitude, latency and topography of the resulting deflections are taken to characterize the underlying mental operations (Woodman, 2010). By comparing ERP components for different stimuli, inferences can be made about different types of non-conscious and conscious responses, such as personal relevance, allocation of attention, expectancy violation, and emotional judgment (Hajcak et al., 2012).

Although EEG is among the most prominent tools for neuromarketing studies, as it allows researchers to capture brain activity at the speed of cognition, it is inextricably connected to some limitations. Despite some commercial claims to the contrary, EEG is not a straightforward methodology. The EEG-based metrics may be challenging to interpret or understand in contrast to direct behavioral measures, such as facial expressions or eye-tracking metrics. Moreover, ERP studies require repeated recordings in order to suppress the unrelated brain activity, constituting the procedure of measuring responses to novel stimuli (e.g., new products or packaging) difficult. Finally, it should be noted that EEG is not an appropriate neuroimaging method for measuring brain activity that originates from regions located deep within the brain, such as the emotional and memory centers. However, the neuroscientific community exhibits notable efforts toward this direction and particularly in the context of EEG-based emotion recognition (Alarcao and Fonseca, 2017).

### 2.2. Eye Tracking

Eye tracking refers to the actual tracking of the movement of the pupil. To this end, a technique called pupil center corneal reflection is typically employed (Guestrin and Eizenman, 2006). The core of this technique lies on a light source (visible or invisible light) that illuminates the eye causing highly detectable reflections and a camera capable of capturing these reflections. By means of suitable algorithms operating on the captured image, the reflection of the light source on the cornea and in the pupil is identified. Finally, the vector formed by the angle between the cornea and pupil reflections can be calculated. Then, by combining the aforementioned vector with other geometrical features, the actual gaze direction can be estimated. Moreover, by employing similar principles, modern eye tracking systems are capable of providing estimations regarding pupil dilation.

Eye tracking is among the non-neural monitoring methods that enable the study of behavioral and cognitive responses without measuring directly the brain activity. Eye tracking provides information about where subjects are looking at, for how long they are looking, the path of the subjects' view and changes in pupil dilation while subjects observe a stimuli (Bercea, 2012). Notably, eye tracking allows measuring the attention focus, hence studying behavioral responses (Laubrock et al., 2007). Typically, eye movement states belong to two distinct categories: fixations and saccades (Zurawicki, 2010). Saccades refer to the type of eye movement where the fovea rapidly moves from one point of interest to another, whereas a fixation describes the condition where the eyes are kept aligned with a target for a certain period of time, allowing for the image details to be processed. The resulting series of fixations and saccades is called a scan path which is used in analyzing visual perception, cognitive intent, interest and salience.

Eye tracking is widely used in combination with electroencephalography allowing practitioners to investigate simultaneously neural activity with respect to particular visual elements during the free viewing of complex stimuli (Baccino, 2011). However, the simultaneous registration of eye movements and EEG is very complicated as it is governed by several data-analytic challenges which have confined the more widespread adoption of this co-registration in neuroscientific research (Dimigen et al., 2011). The main four methodological challenges that have been identified in such hybrid settings, with recent efforts toward overcoming them (Dimigen and Ehinger, 2020), concern: (i) the synchronization of heterogeneous data streams, (ii) the contamination of EEG by ocular artifacts, (iii) the condition-specific temporal overlap between the brain responses evoked by consecutive fixations and (iv) the fact that numerous low-level stimulus and saccade properties also influence the post-saccadic neural responses. Finally, it should be noted that although the artifact-related challenges also apply in uni-modal EEG settings, studies that also include eye-tracking studies typically allow subjects to participate in a free-viewing manner which, inevitably, enhances the ocular artifacts and amplify these problems. Moreover, beyond the ocular activity, in free-viewing modes we anticipate pure neural activity that stems from the brain areas which are related to the eye movement (Kalaganis et al., 2018). As expected, these facts lead to more complex data analysis and sophisticated interpretation procedures.

### 2.3. Electrodermal Activity

The recording modalities previously described are not the sole sources for registering physiological and behavioral responses. Another non-invasive physiology monitoring tool is the electrodermal activity (EDA), historically also known as Galvanic Skin Response (GSR), that identifies alterations in the sweat gland activity. The most common measurements for EDA-related activity are the skin conductance levels (SCL), the short-duration skin conductance responses (SCRs), and the number of GSR peaks, that are mainly employed to derive information regarding the emotional arousal. Nevertheless, there are also cases in which the aforementioned indicators have been employed for monitoring workload and tracking attention (Hernández-García et al., 2017).

GSR in the area of neuromarketing is employed as a tool to analyze the corresponding activity with the aid of signal analysis algorithms that result in various emotional indices (EI) capable of determining the consumer's emotional arousal (Białowas and Szyszka, 2019). It is important to note here, that since both positive and negative stimulation can induce increased arousal levels and therefore increased conductance levels, the GSR-provided information reflects only the intensity of the elicited emotion and not its exact type (e.g., happy, sad). This is probably the reason that GSR is rarely included in studies as the sole physiological modality and is usually accompanied by other modalities, like EEG.

### 2.4. Electrocardiography

Electrocardiography measures the changes in the electrical activity of the heart, by appropriately placing electrodes on the participant's body. The most common metric regarding this modality is the Heart Rate (HR), that in essence measures the number of the heart's beats/contractions over a period of time equivalent to 1 min, hence its measurement unit is the bpm (i.e., beat per minute). The heart rate variability (HRV) identifies the alterations in the speed of the heartbeats that can be attributed to various factors including but not restricted to stress, environment, psychological status, or emotional status. Given that such physiological responses can among others objectively indicate ones feelings, much work has been done in the area of emotion recognition using ECG as a means to measure HRV.

Unlike EDA, HR can also be used to classify the emotion type and not only its intensity with several papers reporting the correlation among HR and emotional valence (e.g., Mather and Thayer, 2018). More specifically, when the observed levels of HRV are low the arousal levels are expected to be increased, whereas high HRV levels are linked with decreased arousal levels. Nevertheless, considering that both modalities are employed to determine the emotional arousal, several studies examine them simultaneously considering the complementary nature of the provided information (e.g., Affanni, 2020). Within the context of neuromarketing, the most typical approach is to quantify the HRV to determine the emotional responses resulting in various emotional indices (Appelhans and Luecken, 2006), that are often combined with the ones derived by EDA.

### 2.5. Facial Expressions

A facial expression refers to the motion or the positioning of the muscles lying beneath the skin of the face. According to one set of controversial theories, these movements convey the emotional state of an individual to observers. The facial expressions of humans serve as a dynamic tool for non-verbal communication and is among the primary means of conveying social information between humans. Facial expressions fall into two categories: (a) voluntary and (b) involuntary where each one associated with different neural origins. The former is often socially conditioned and follows a cortical route in the brain whereas the latter is believed to be innate and follows a subcortical route in the brain. Since the decoding of facial expression is a particularly important task in several domains (including neuromarketing) as it may uncover emotions and provide information concerning the response in various perceptual cues (e.g., a positive smile or a negative frown), the corresponding literature is rich of methodologies for automated decoding. Among these methods, the field is dominated by facial electromyography and computer vision-based decoding techniques.

Facial electromyography (fEMG) refers to the electromyography monitoring technique that measures the facial muscles' activity. It actually detects and amplifies the tiny electrical impulses that are generated by muscle fibers. The two major facial muscle groups that fEMG primarily focuses are: (i) the corrugator supercilii group which is associated with frowning and (ii) the zygomaticus major muscle group which is linked with smiling. On the other side lie the computer vision-based approaches that exploit advances in camera technology and image processing algorithms. Although the employed algorithms are much more complicated than the ones used in fEMG studies, the scientific community has managed to achieve significant outcomes capable of identifying the emotional facial expressions in near real-time (Canedo and Neves, 2019). The core of computer vision algorithms lies on initially detecting and then semantically decoding facial landmarks.

Although involuntary facial movements occur when a person experiences emotional states and facial expressions are typically considered to be consistent across cultures, we should note that, facial expressions have been evolved so as to communicate emotional states in social context (Fridlund, 2014) and therefore occur at relatively low levels in the context of passive media perception upon which the vast majority of marketing content relies (e.g., brochures, television, internet, etc.) (Smith and Marci, 2016). Beyond the aforementioned drawback, facial expression decoding exhibits similar challenges, with respect to its co-registration with EEG, as the eye-tracking. Typical EEG studies ask participants to refrain from producing any facial expression as the produced electrical activity is known to highly contaminate the EEG signals. In conjunction with the need to synchronize these heterogeneous data-streams it becomes apparent that these settings lead to demanding and complex experimental protocols.

## 3. Past Decade Neuromarketing Trends

Fueled by early success stories, the neuromarketing domain advanced rapidly during the last 10 years. As exciting new techniques were being adapted from medical research to the commercial domain, many neuroscientists and marketing practitioners have taken the chance to exploit them so as to uncover the answers of the most important marketing questions. However, they sometimes failed to recognize that each one of the various monitoring methods is accompanied by particular limitations. Therefore, the most appropriate tool to employ should be dictated by the nature of the problem to be solved and the question to be asked. As this fact was gradually being realized, the hybrid approaches started to enter in the picture. In an effort to alleviate the shortcomings of a single modality and answer more complicated questions neuroscientists and other research practitioners, nowadays, focus more on studies that combine multiple monitoring methods.

**Figure 2:**
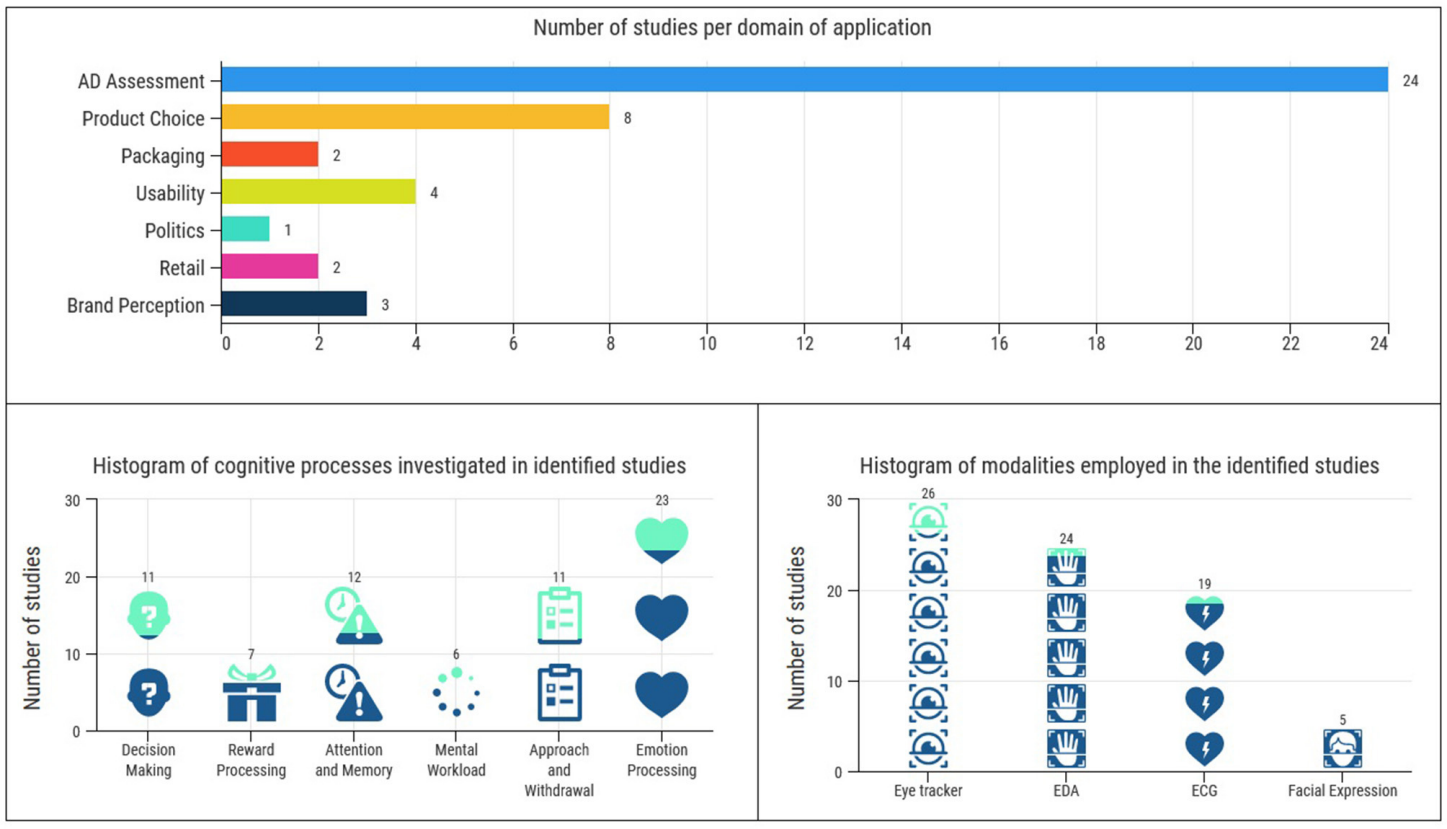
**(Top)** The distribution of identified studies based on categorization with respect to the domains of applications. **(Bottom left)** Histogram indicating the cognitive processes that the identified studies investigate (overlapping). **(Bottom Right)** Histogram indicating the, complementary to the EEG, modalities employed in the identified studies (overlapping).

With the scope to cover this promising neuromarketing approach, we present all the identified neuromarketing papers of the last decade that employ hybrid EEG settings. The papers have been categorized by means of application domain in order to group together efforts that address a common issue and uncover common underlying trends in neuromarketing research. Subsection 3.1 is devoted to efforts toward evaluating advertisements which is the most dominant application domain as the vast majority of the identified studies falls into this category. Subsection 3.2 is dedicated to product choice/preference. Subsection 3.3 is dedicated to product and brand perception and includes usability studies as well as packaging and brand perception. Finally, subsection 3.4 concerns other application domains which include retail studies and politics. For each work discussed, beyond explaining its main findings, we provide evidence about the cognitive processes it investigates. Figure 2 presents in the upper panel the non-overlapping distribution of the identified works over the domains of application and, the overlapping distribution over the variety of modalities and the cognitive processes of interest (e.g., some studies investigate more than one cognitive processes or employ more than two complementary to the EEG modalities) in the bottom left and right panels, respectively.

A short description of the adopted methodology for detecting all the relevant studies is in order. First, we initiate a bibliographic search based on Google Scholar using the keywords “neuromarketing” or “EEG/electroencephalography” as query. This resulted in 3,500 publications. Then the studies that were either in non-English language or were not published in a peer-reviewed venue were discarded. This procedure eliminated about 2,500 works and left ~1,000 for a second assessment round. During this second round, judging the studies by their title, abstract and, where necessary, the whole document we identified the works that combined EEG with another modality in neuromarketing. This lead to a total amount of 43 studies that are included in our survey. We note that, although we put significant effort into identifying all hybrid EEG neuromarketing studies of the last decade, the possibility that some studies may have gone unnoticed remains open. This work is accompanied by a Supplementary Material that contains extensive information for each one of the selected studies.

### 3.1. Advertisement Assessment

Creating appealing advertisements (ads) that will convince consumers to purchase a product or service is of paramount importance in the area of marketing. Therefore, the process of advertisement assessment is critical in the advertisement creation pipeline, as it can provide insights regarding the ad's expected impact. The advertisement's impact is based on various factors, like the intended customers and their value of decision or the type of the offered product or service (Glowa, 2002). Nevertheless, if the aforementioned factors are predetermined, the evaluation of its effectiveness can be achieved via a series of metrics that belong to the broad area of neuromarketing, including among others emotional processing, decision making, attention and memory, approach and withdrawal motivation, mental workload, and reward processing with the scope of evaluating its efficacy.

Emotions are known to significantly affect the decision making process and therefore several ads are designed with the scope of eliciting specific emotions to potential customers. Consequently, the goal of advertisement assessment via neuromarketing is 2-fold: firstly to determine whether the intended emotion is elicited and secondly to identify other elicited emotions. In one such study, Matukin et al. (2016) used EEG and ET to identify the users' emotions when static images of DVD covers with and without awards information were presented to them. The analysis of the pre-frontal asymmetry and of the gaze spatial information among 40 individuals indicated that in the case of covers with awards information the emotional involvement was systematically increased. Balanzó et al. (2011), Neomániová et al. (2018), and Emsawas et al. (2019) used audiovisual advertisements (i.e., spots) instead of static ones with the scope of improving the designed ads based on the users' physiological responses. The first two studies aimed at the identification of scenes characterized by low or negative emotional engagement that usually indicates low user interest, and therefore, should be redesigned appropriately. The identification of such parts was performed with the aid of physiological responses that in the first case were EEG and FE while in the second EEG, ET, and GSR. In the latter study, EEG, ET, and EOG metrics were translated to a total “feeling score” with its negative values being associated to negative emotions. The most characteristic example of the aforementioned association was the presentation of a disgusting scene that resulted in the lowest values of the “feeling score.”

Emotions also have a direct impact in other cognitive processes including working memory and attention, approach and withdrawal motivation and mental workload, hence their examination is often performed in parallel. Grimaldi (2012) jointly examined emotional responses with working memory and attention with the scope of identifying weak points in advertisement spots, that should be redesigned or removed from the specific spot. The formulated attention and memory indices delivered upon EEG, ET and GSR signals reliably detected the scenes characterized by low levels in both interest and emotional involvement. Vecchiato et al. (2014b) used the same indices and showcased that Eastern TV commercial is perceived in a more pleasant fashion compared to the Western one. Colomer Granero et al. (2016) combined the aforementioned indices with GSR and HR measurements to classify advertisement spot into three categories (i.e., positive, neutral, and negative), with the use of the selected physiological features resulting in correct classification of the spot in nine out of ten times.

Vecchiato et al. (2014c), Piwowarski et al. (2019), and Mateusz and Kesra (2020) examined the approach and withdrawal motivation alongside with emotional responses, working memory and attention. All three studies analyzed the physiological responses of EEG, GSR, and HR by formulating emotional, memorization and approach-withdrawal indices, with the scope of investigating the effectiveness of audiovisual advertisement. Piwowarski et al. (2019) identified a lack of interest for a specific social advertisement that was attributed to the low levels of the entirety of the indices for the majority of the 30 participants of the study, indicating that the advertisement should be redesigned. Similarly, Mateusz and Kesra (2020) used the same metrics to identify specific ad scenes in fragment advertisement that were characterized by indices of low values in order for them to be redesigned for increasing the ads' effectiveness. In the last case, Vecchiato et al. (2014c) examined the differences in the perception of TV ads among genders, with the aforementioned indices indicating that the same ads do not have the same effect on the two genders (e.g., brand and product of interest).

The combination of emotional assessment with approach and withdrawal motivation is also examined by some studies. Vecchiato et al. (2014a) used approach/withdrawal and emotional indices to determine whether an ad is liked or not by a total of 15 individuals that participated in the study with the employed modalities being EEG, GSR, and HR. Cartocci et al. (2016) added to the indices described in Vecchiato et al. (2014a) the effort index to evaluate the effectiveness of antismoking campaigns. The effectiveness of social advertisement was further analyzed by Cartocci et al. (2017) and Piwowarski (2017) that considered the mental workload as an extra cognitive process that could affect the evaluation mechanism.

Another component commonly examined alongside emotional assessment is the mental workload. In one such study, Martinez-Levy et al. (2018) examined the effectiveness of product placement in music videos, using emotional and mental effort indices (formulated from EEG, GSR, and HR measurements) combined with the number of fixation (derived from ET). Their results indicate increased measurements in the emotional and mental effort indices for videos with product placement and that the number of fixations is correlated with the recall process. Michael et al. (2019) used EEG and ET measurements to understand the cognitive processes involved in the selection of a travel destination, showing that different destinations trigger different responses both in the emotional and mental workload metrics.

The single use of the aforementioned cognitive processes or a combination of them without including emotion recognition is rarely anticipated for purposes of advertisement assessment. More specifically, Cherubino et al. (2016) used approach/withdrawal indices derived upon EEG, GSR, and HR metrics to identify differences in ad perception among adults and young people. The indices indicated that younger ages were mainly attracted by the funny scenes of the clip while the older ones by the story-telling ones. García-Madariaga-Madariaga et al. (2020) validated with the use of mental workload indices based on EEG and GSR and the duration of fixations estimated using ET, that advertisements of complex visual stimulations increase the mental workload and the time required to grasp the conveyed by the advertisement message. Decision making and reward processing are combined in the studies of Vecchiato et al. (2013) and Dimpfel et al. (2015) that used these two metrics to evaluate TV advertisements. In the first case, EEG, GSR, and HR measurements were merged to separate among two indicative classes of “like” and “dislike” regarding the displayed advertisements, while the second identified among five different bank commercials the one with the highest mental activation using both EEG and ET measurements. Finally, the attention/memory process was examined in parallel with the decision making process by Ural et al. (2019) that used EEG, ET, GSR, and HR as a means to separate the stimuli preceding an advertisement and the ones that are part of it to determine the visual attention/memory and the decision making process.

Finally, the event related potentials (ERPs), a popular approach within the neuroscientific community has also been employed for ad assessment purposes. An ERP is a brain response elicited upon a stimulation that is mainly examined to determine selective attention and perception and can be easily captured via EEG. Samsuri et al. (2016) included a combination of ERPs and gaze heatmaps to determine the level of attention when car photos of different viewpoints were presented to a total of 15 participants, with the performed statistical analysis revealing higher attention levels when left side images were displayed. A study in the field of car advertisement was also performed in Bharu (2016), with the scope of providing design guidelines to marketers about presentations and price placements using the toolkit described in Samsuri et al. (2016).

### 3.2. Product Selection

Product selection is a key aspect of marketing, and it has been extensively studied in neuromarketing with the scope of identifying the key elements of a product that will lead to its purchase. It is a process that inherently depends on decision making and, hence, several studies have examined it. In one such study, Khushaba et al. (2013) investigated the physiological decision processes during decision making for the task of cracker selection, with the aid of an EEG and an ET device. In their study, illustrations of several crackers that were different in terms of shape, topping and flavor were provided to a total of 18 participants with phase locking value (PLV) and the total time spent in each being used as the metrics for EEG and ET, respectively. The preferred crackers (as reported by the participants) were characterized of both significant changes in the frontal and occipital area and increased time spent observing the specific illustration. In the same direction, Garczarek-Bak (2018) with the aid of the same modalities (i.e., EEG and ET) compared the product selection process between products of different private labels (PL) in a group of 16 individuals, with the left frontal asymmetry predicting an affirmative purchase decision in several cases, while the ET measurements did not reveal any differences between men and women regarding the aesthetics of the PL products. A by-product of this study was that the product selection process was invariant to the price of the PL products.

Emotions also play a critical role in the product selection process, with Huseynov et al. (2019) highlighting the correlation between negative emotions and the purchase intention using a mixed prediction model consisting of EEG, ET, and FE metrics that systematically outperformed the single modalities. On the contrary, Horska et al. (2016) found that positive emotions (i.e., happiness and surprise) were highly correlated with high scores obtained in the subjective evaluation of various wines, indicating that the possibility of a purchase is increased when the positive emotions are elicited. In this case, the emotional state was separately examined using EEG measurements and FE, with both metrics yielding in similar responses regarding the wine selection process. Almomani et al. (2019) examined in a similar fashion the impact emotions have in the selection of a movie by also including ET and GSR measurements, with the designed emotional model being able to accurately predict user's choices. Bettiga et al. (2020) compared the emotional responses between functional and hedonic products with the aid of several modalities (i.e., EEG, ET, HR, and GSR) in a sample of 21 participants, with the employed physiological metrics, indicating that both functional and hedonic products elicit similar emotions to the participants and that there is no statistical difference between the emotions.

Finally, the product selection process is highly affected by the product's ability to attract consumers, as it is highly unlikely for an individual to select a product that is not perceived as appealing. With this hypothesis in mind, various research groups have examined the (neuro)physiological responses with respect to approach and withdrawal motivation. In particular, Christoforou et al. (2017) examined the potential success of a movie based on whether its trailer was appealing to a total number of 27 participants. The use of approach-withdrawal indices based on EEG (i.e., the instantaneous powers of beta and gamma bands) and ET (i.e., the attentional asymmetry) metrics, could reliably predict the commercial success of a given movie by directly comparing them with key performance indicators. Garczarek-Bak and Disterheft (2018) compared private labels with national brands with frontal asymmetry in alpha, beta, and gamma bands aiding again in the prediction of purchase decisions that were highly correlated with the brand's publicity.

### 3.3. Product and Brand Perception

Another important aspect of marketing focuses on the product itself and the dynamics it creates in the market. Products are usually classified as physical and non-physical, with the second category being widely known as services. There are various attributes that are known to affect the market value of a specific product/service, with the most important ones being package, usability and the overall brand perception. Packaging is a notion that is usually anticipated in the case of physical products, usability mainly describes the non-physical ones, while brand perception can be encountered in both.

Packaging is known to be one of the core elements of marketing that often has a direct impact on the brand perception, as a package that attracts the consumer's attention is more likely to be selected and consequently be purchased. Within the concept of designing an attractive package, Al Pop et al. (2013) provided guidelines for the design of honey packages using the physiological responses of EEG, ET and GSR with their main focus being on elements that increased the attention and also elicited positive emotions to a total of 44 participants. In a similar fashion, García-Madariaga et al. (2019) examined different packaging attributes, namely images, texts and colors, with the scope of identifying the ones that would lead to increased engagement levels to the 40 participants of the study using EEG and ET.

As previously mentioned brand perception is often affected by the product's packaging, with dos Santos et al. (2020) using EEG, ET, GSR, and HR to determine the impact different logo designs and placements have on the consumers' (emotional) engagement and approach/withdrawal motivation. Their study indicated that specific logo redesigns and placement could have a positive impact on both consumer engagement and approach/withdrawal motivation. On the contrary, the object of study for Sung et al. (2019) and Sargent et al. (2020) was to compare market-leading brands with local/non-luxury brands, with both studies using mainly emotional indices to perform the comparisons. In the first case, EEG, GSR, HR, and FE were employed as a means to determine the consumers' emotional engagement, with products of the non-luxury category being characterized by positive emotions, while the market-leading ones illustrated higher values in the approach/withdrawal indices. In the second case, the emotional valence derived upon EEG and GSR was in higher levels for the leading brand for several of the study's 26 participants, with the specific response being considered indicative regarding the participants' preference.

Finally, usability quantifies a system's capacity to be user friendly and consequently in the majority of the cases refers to services. Dimpfel and Morys (2014) and Kvasnicova et al. (2016) examined the usability of web sites using EEG and ET devices. The first study employed EEG spectral features and gaze heatmaps to evaluate the web site of five banking institutions and identify the one with the highest usability levels, while the second identified the components of a specific web site that attracted users the most. Adhami (2013) examined the usability of mobile applications, using emotional and attentional indices derived from EEG and ET, respectively to recognize the visual elements that were more appealing to a total of 30 participants. With the aid of the same modalities (i.e., EEG and ET) (Gonzaga et al., 2016) explored user perception and attention in Facebook organic posts, showing that posts characterized by high attention levels are more likely to reach to more individuals.

### 3.4. Other Application Domains

Besides the aforementioned application domains that stand out in the majority of neuromarketing studies, a limited number of studies also focuses on other domains. More specifically, Vecchiato et al. (2014d) employed the physiological responses of EEG, GSR, and HR to investigate the trustworthiness of images illustrating political candidates, with graph and power spectrum components being identified as features that were highly correlated with the judgment of trustworthiness and that could therefore be considered indicative regarding the vote prediction. Cherubino et al. (2017) performed an on-field evaluation of the different departments in a retail store using portable EEG and ET devices. More specifically, the emotional and attentional indices that were used in this study, revealed that the fruit and vegetable department of the store was the one with the highest emotional and engagement levels and therefore has the potential to attract the consumers' attention. In addition, the study demonstrated that packaging with specific graphical illustrations (e.g., real faces) may serve as a useful tool to elevate the level of pleasantness and emotion; hence, capture the consumers' attention in order to make department more engaging as well. In a similar on-field evaluation, Gonchigjav (2020) identified the wine section as the most appropriate one to present promotional installations.

## 4. Discussion and the Future of Neuromarketing

Neuroscience technology has entered the commercial sphere during the last decade by developing wearable and ergonomic devices that allowed researches to investigate neural responses not only *in vitro* but also *in vivo* (e.g., daily life) environments. Therefore, it is now possible to record and study consumers' actual cognitive responses (e.g., emotional reactions) without asking questions or interfering with the task of interest. In contrast to typical marketing practices (e.g., questionnaires, response times, behavioral performances, etc.), neuromarketing approaches allow researchers to collect information in real time and in a continuous manner. Neurotechnology enables the measurement of the user's actual mental and emotional condition in an objective fashion and therefore paves the way for a wide range of applications. However, there are still a lot of challenges that should be addressed before the field thrives further.

Toward this goal, in 2015 a key study (Plassmann et al., 2015) identified three major challenges that the field faces: First, the vast majority of studies are limited to providing only correlational but not causal evidence and therefore end up facing criticism. In other words the first challenge is related to the fact that consumer neuroscience research is restricted to studying consumers' brain instead of consumers' behavior. In order to overcome this obstacle, marketing researchers should consider consumer neuroscience as a method to improve the way behavioral measures are obtained and interpreted instead of a means to replace traditional approaches. The second challenge concerns the interpretation of findings. Very often, neuromarketing studies are based on the assumption that a brain region is inextricably (i.e., causally) connected to a cognitive process. To put it differently, researchers often conclude that participants exhibit a particular psychological process based on the observed neural activation in a particular brain region. Although reverse inference is problematic for any research that links neuroscience to behavior, this obstacle can be overcome by relying on theory-driven approaches for designing studies and by applying robust statistical tools for result interpretation. The last challenge stems from the considerably smaller sample sizes than those used in traditional psychological/behavioral research studies which leads to questionable findings. By employing small sample sizes several issues arise: (i) neuroscientific findings are unreliable, (ii) neuroscientific findings cannot be generalized to a wide population, and (iii) the possibility of opportunistic findings remains widely open. Neuromarketers may focus on smaller experimental protocols and aim to more targeted research questions in order to investigate larger populations in shorter amounts of time.

Although the aforementioned challenges mostly concern the research discipline of neuromarketing, several existing practices also limit its commercial and industrial application. In 2019, the “Neuromarketing Science and Business Association” performed a survey^1^ so as to understand the challenges that the commercial aspect of neuromarketing faces. Two main themes appeared, revolving around credibility and trustworthiness. More specifically, they concern: (i) the readiness of clients to adopt neuromarketing solutions, and (ii) the reputational risk arising by inexperienced or under-qualified vendors (e.g., the negative effects of over-promising and under-delivering services). Although the methods applied nowadays have a more sound scientific basis, neuromarketing still straggles to prove its credibility. This is attributed to the fact that too many providers entered the neuromarketing field in its early days and failed to apply sound and robust scientific methods.

In addition to questioning the validity of neuromarketing practices, the field is accompanied by ethical criticism. Is neuromarketing an approach of understanding and defining the human behavior or a lurking method that leads to unethical manipulation of consumers and potentially discover the much wanted “buy button” in their brains? Actually, neuromarketing revolves around shaping the nature and appeal of products. Typically, neuromarketing studies present findings that rely on brain data obtained from several people who voluntarily participate in experiments that take place in constrained laboratory environments. The combination of behavioral and neural data is indeed powerful; hence, the potential for exploitation remains a practical possibility. However, neuromarketing is not about exploiting brain signals and invading personal privacy by inferring individual intentions and motivations at the speed of cognition. Currently there is no evidence for a “buy button” in the brain; the concept is closer to hype than to reality, and the controversy is mostly governed by concerns related to the practical effectiveness (Illes and Mizgalewicz, 2012). Nonetheless, as the (neuro)marketing field advances and worrisome research findings (Dezfouli et al., 2020) see the spotlights of publicity, regulations and policies should be developed so as to mitigate potentially unethical exploitation practices.

The field of neuromarketing is expected to gain additional popularity, despite these challenges and concerns. However, on the basis of disseminating the variety of information, a comprehensive theory should be developed that will combine individual internal states with general social processes (e.g., word of mouth, imitation, and other social phenomena) as measured by means of physiological monitoring methods (Parkinson et al., 2018). Such kind of theory is apparently missing and may reflect the most promising research direction from a theoretical and scientific perspective. Stronger collaboration between scientists -and their corresponding scientific tools- stemming from a wide variety of complementary disciplines (e.g., neuroscientists, economists, advertisers, and sociologists) may become more common in the coming years. Such a collaborative environment will hopefully drive the field to uncovering the subtle and perplexing nature of human decision making in real circumstances.

However, studying human brain dynamics in natural environments is an extremely difficult task. Existing brain imaging approaches are not designed to reliably support such studies in realistic scenarios. Conventional neuroimaging studies treat the electrical activity generated by the eyes or muscles during physical movements as artifacts that should be avoided as they contaminate the signals of interest. This perspective has led to experimental protocols that confine participants' mobility, even in tasks that involve movement (Makeig et al., 2009; Gramann et al., 2011). These constraints may possibly change the way information is perceived and processed by a human (Gramann, 2013).

On the other hand, the embodied cognition paradigm claims that the body's interaction with the world is the basis of cognitive processes (Wilson, 2002). Under this viewpoint, when studying cognitive processes and their neural basis, both perception and action should be taken into account. Toward this direction, upcoming advances in wearable sensor technology and brain imaging may provide exciting opportunities to develop new multimodal neuromarketing approaches. All these technologies will possibly allow us to design new paradigms by integrating information from different sensors, such as EEG, ET, cameras, wearables, etc. This vast amount of information could give us the ability to study consumer behavior in natural environments and everyday settings following an holistic approach.

As cross-disciplinary researchers try to integrate neuroscientific tools to traditional marketing practices, it keeps becoming more evident that the existing neural recording methods alone cannot appropriately answer the major research questions. Each of the techniques used in neuromarketing research is accompanied by particular strengths and weaknesses and these attributes constitute them more or less suitable for different research situations. Hence, the research questions and objectives should drive researchers to choose the particular combination of modalities that will lead to more effective neuromarketing studies and strategies capable of providing more accurate answers to the questions posed.

## Author Contributions

SN, VO, and IK conceived the study. FK and KG collected and processed the material and drafted the paper. SN, VO, and NL supervised the meta-analysis. NL, SN, and IK offered the critical revisions. All authors reviewed the manuscript.

## Conflict of Interest

The authors declare that the research was conducted in the absence of any commercial or financial relationships that could be construed as a potential conflict of interest.

## Footnotes

^1^https://nmsba.com/buying-neuromarketing/the-state-of-neuromarketing-in-2018.
